# Differential metabolic responses in breast cancer cell lines to acidosis and lactic acidosis revealed by stable isotope assisted metabolomics

**DOI:** 10.1038/s41598-020-78955-2

**Published:** 2020-12-15

**Authors:** Jiayue Gao, Zhiying Guo, Jianhua Cheng, Bo Sun, Jie Yang, Haijing Li, Shengming Wu, Fangting Dong, Xianzhong Yan

**Affiliations:** 1 National Center of Biomedical Analysis, No. 27 Taiping Road, Beijing, 100039 China; 2 Hepatal-Biliary-Pancreatic Center, Beijing Tsinghua Chang Gung Hospital, Beijing, 102218 China

**Keywords:** Biochemistry, Cancer

## Abstract

Extracellular acidosis is considered as a hallmark of most human tumors, which plays an important role in promoting tumor malignant and aggressive phenotype in tumorigenesis. Acidosis and lactic acidosis can induce different responses in tumors. Previous studies have associated the response to lactic acidosis of tumors with good survival outcomes. In this study, we investigated the metabolomic changes in triple negative and luminal subtype breast cancer cell lines in response to acidosis and lactic acidosis. Our results showed that acidosis results in the reduction of cell viability and glycolysis in breast cancer cells, which is reversely correlated with the malignancy of cell lines. Under lactic acidosis, this reduction is reversed slightly. Untargeted metabolomic profiling revealed that glutaminolysis and fatty acid synthesis in cancer cells under acidosis are increased, while TCA cycle and glycolysis are decreased. Under lactic acidosis, the pentose phosphate pathway and acetate release are increased in MDA-MB-231 cells. The current results uncovered the different metabolic responses of breast cancer cells to acidosis and lactic acidosis, demonstrating the power of combined untargeted and stable isotope assisted metabolomics in comprehensive metabolomic analysis.

## Introduction

Solid tumor is facing a complex microenvironment characterized of hypoxia and acidification^1^. These changes are caused by a combination of abnormal tumor vasculature, rapid proliferation and altered metabolism. Metabolites in turn can alter the relative gene expression, epigenetic modification, sequentially reprogram the metabolism of cancer cells^2–4^. Heterogeneity of tumor tissues makes tumor microenvironments more complex^5^. Hypoxia has been extensively studied, whereas much less is known about the cellular response to acidosis, how they metabolically adapted to acidosis.

More than 90 years ago, Otto Warburg noticed that cancer cells obtain energy via glycolysis, in which glucose is metabolized to lactate rather than enter into TCA cycle, even under aerobic conditions^6^. Under hypoxic condition, the glycolysis was even more active, together with much more lactate production^7^. Lactate is transported to extracellular by monocarboxylate transporters along with protons which give rise to medium acidification^8^. Additionally, cancer cells may pump protons out to the extracellular to maintain a relative alkaline intracellular pH through many ways, which include co-transport of protons and negative ions (bicarbonate ions, acetate ions), CO_2_ hydration, exchange of hydrogen ion and cation^9–12^. Acidic microenvironment is nowadays considered as a hallmark of most human tumors^13,14^. It is characteristic of tumor microenvironment that the acidic pH, or called extracellular acidosis, ranging from 5.8 to 7.1^15–18^. Extracellular pH in a wide array of cancers has been determined more acidic than the normal tissues, which may decrease as the tumor size increases. With the external pH of tumors increased, the spontaneous metastasis is getting reduced^19^.

Previous studies have suggested that lactate is not only the major energy providing form^20^ but also the factor that can drive the reprogramming of metabolism in cancer cells^21,22^, including glycolysis, tricarboxylic acid (TCA)^23^, glutamine metabolism^24^ and fatty acid metabolism^25^. Acidosis could decrease oxygen and glucose consumption and also decrease lactate accumulation^26,27^ to suppress glycolytic metabolism. Acidosis elevated the concentration of acetyl-CoA via inducing fatty acid oxidation (FAO), which satisfy the demands of TCA pathway. The expression and activity of glucose-6-phosphate dehydrogenase (G6PD) was also increased in acidosis, which enhanced oxidative phase of pentose phosphate pathway (PPP) activities^21^. It is reported that lactic acidosis will induce much more alternations of genes than hypoxia. These genes involved in cell cycle, cell proliferation, glycolysis^28,29^, hypoxia responses, and autophagy^30–32^. Lactate accumulation in tumors, the concentration of which can reach as high as 40 mmol/L^33^. For non-cancerous cells, the extracellular acidic microenvironment is harmful, whereas acidification plays an important role in promoting tumor malignant and aggressive phenotype in tumorigenesis^34–36^. Therefore, it is important to investigate the influence of acidosis on the tumor metabolism and to understand the contributions of lactate in this process.

Breast cancer is a malignant tumor that is common in female population^37^. Different treatment schemes are adopted based on the classification of tumors^38,39^. MDA-MB-468 and MDA-MB-231 are basal-like and triple negative cell lines with high invasiveness, whereas MCF-7 and SkBr3 are luminal subtype cell lines with low invasiveness. It was reported that cell lines with different malignant grade have different responses to acidic stimuli^40^. Here, we reported a comprehensive metabolic view of breast cancer cell lines under acidosis and lactic acidosis, which help to compare metabolism in cell lines with different degrees of malignancy. These data help us to understand the effect of acidic environment on cancer cells from the point of view of cell metabolism, which are helpful for developing therapeutics.

## Results

### Cell viability

**Figure 1:**
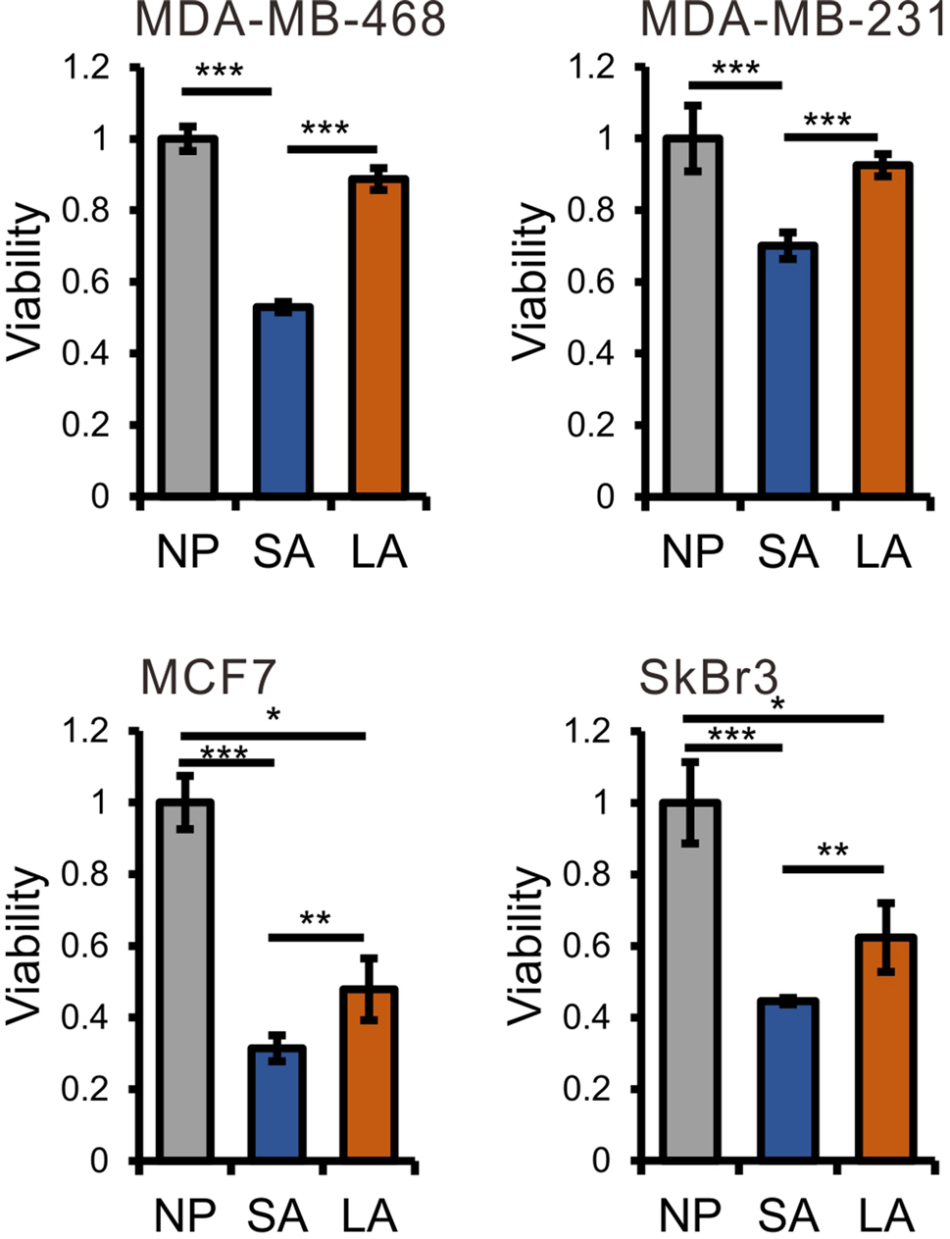
Lactate acidosis can reverse the decrease in viability of breast cancer cells induced by acidosis (*NP* normal pH groups, *SA* acidosis groups, *LA* lactic acidosis groups). Values are expressed as mean ± SD. *P < 0.05, **P < 0.01, ***P < 0.001 by unpaired Student’s test, n = 6.

The effects of acidosis on the viability of breast cancer cell lines (MDA-MB-468, MDA-MB-231, SkBr3 and MCF-7) were assessed by using CCK-8 assay. As shown in Fig. 1, acidosis resulted in significant decrease of viability of all four cell lines, especially MCF-7 and SkBr3, which can be ameliorated by the addition of lactate under lactic acidosis. MDA-MB-468 and MDA-MB-231, both of which are basal-like and triple negative cell lines^41^, are less susceptible to acidosis, and lactate could recover the decreased viability to near that under normal pH condition. On the other hand, MCF-7 and SkBr3, both of which are luminal subtype cell lines^41^, are more susceptible to acidosis and lactate can only partially restore the viability. Subtle difference in cell viability between these two cell lines was also observed. The SkBr3 cells is less susceptible to acidosis than MCF-7 cells.

### The influence of acidosis on glycolysis

**Figure 2:**
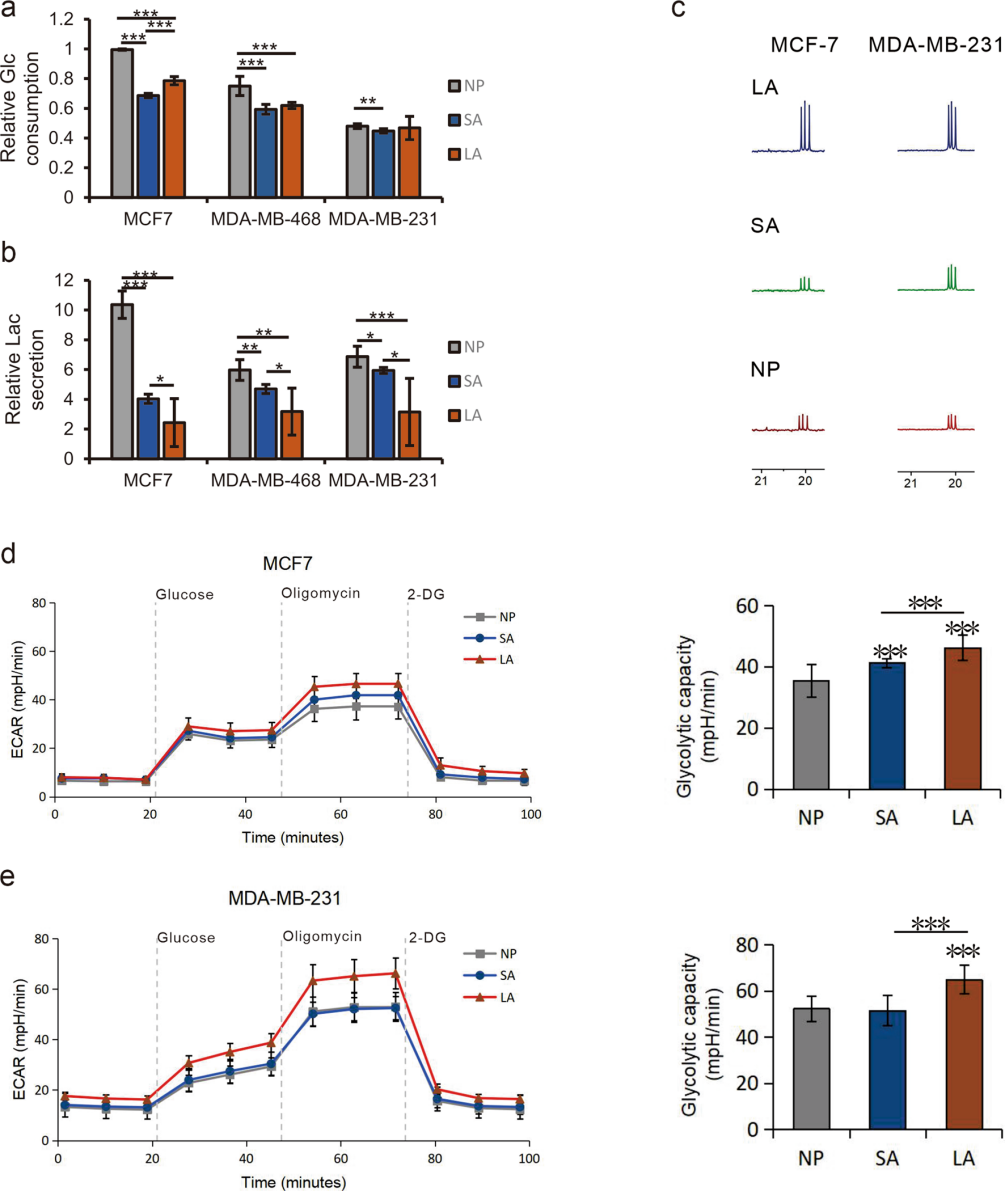
Acidosis can decrease the glycolysis in cancer cells. Glucose consumption (**a**) and lactate excretion (**b**) of breast cancer cells under different culture conditions. ^13^C NMR spectra of labeled lactic acid in MCF-7 and MDA-MB-231 cell lines (**c**). The MCF7 (**d**) and MDA-MB-231 (**e**) cells were detected for ECAR after treated for 48 h. Values are expressed as mean ± SD. *P < 0.05, **P < 0.01, ***P < 0.001 by unpaired Student’s test, n ≥ 6.

It has been reported that acidosis can decrease glycolysis of cancer cells^28^. Therefore, we compared the glucose consumption and lactate production in culture media under acidosis, lactic acidosis and normal pH. The relative content of glucose consumed and lactate excreted over 48 h was quantified from ^1^H NMR spectra. The results showed that the glucose consumption in MCF-7, MDA-MB-468 and MDA-MB-231 cells was decreased significantly under acidosis (SA groups) compared with that under normal pH (NP group), especially for MCF-7 cells. Under lactic acidosis (LA groups), this decrease was reversed in MCF-7 cells significantly, as well as in MDA-MB-468 and MDA-MB-231 cells slightly (Fig. 2). The glucose consumption (Fig. 2a) in MCF-7 and MDA-MB-468 cells under lactic acidosis is still significantly lower than that under normal pH, whereas in MDA-MB-231 cells the consumption is close to that under normal pH. This tendency of changes in glucose consumption is consistent with that of cell viability. On the other hand, the production of lactate (Fig. 2b), the terminal metabolite of glycolysis, was also significantly decreased in all three cell lines under acidosis, with the most drastic change in MCF-7 cells. Under lactic acidosis, the production of lactate was further reduced as compared with that under acidosis, in contrast to the changes in viability and glucose consumption. This may be due to the increased TCA activity which consumes pyruvate through the production of acetyl-CoA (Ac-CoA), or the increased utilization of lactate by cancer cells under lactic acidosis. These results showed that acidosis can decrease the glycolysis in cancer cells. We performed ^13^C NMR experiments on the extracts of MCF-7 and MDA-MB-231 cell lines, cultured in media supplemented with a mixture of U-^13^C_6_-Glucose and 1-^13^C-Glucose (1:1). As shown in Fig. 2c, the spectra results showed that the intracellular labeled lactic acid increased obviously in lactic acid group which illustrated that lactic acidosis can alleviate inhibition of glycolysis caused by acidosis. The response to acidosis in two triple negative breast cell lines is much weaker than in MCF-7. The extracellular acidification rate (ECAR) is widely used to evaluate the progress of glycolysis. The increased ECAR of cells cultured in lactic acidosis group indicates that lactic acidosis could promote glycolysis (Fig. 2d,e), which is consist with the ^13^C NMR results.

### Untargeted metabolic profiling of acidosis and lactic acidosis response in breast cancer cells

We evaluated the metabolomic profiles of breast cancer cells cultured in acidosis condition (pH 6.5), lactic acidosis condition (pH 6.5 with 10 mmol/L lactate) and normal condition (pH 7.4).

Representative total ion chromatograms (TIC) of intracellular extracts from MDA-MB-231 and MCF-7 cells are shown in Supplementary Fig. S1. Multivariate statistical analyses were conducted on these data sets. Partial least squares discriminant analysis (PLS-DA) indicated that the metabolic profiles of each cell lines at variant culture conditions can be apparently separated (Supplementary Fig. S2).

**Figure 3:**
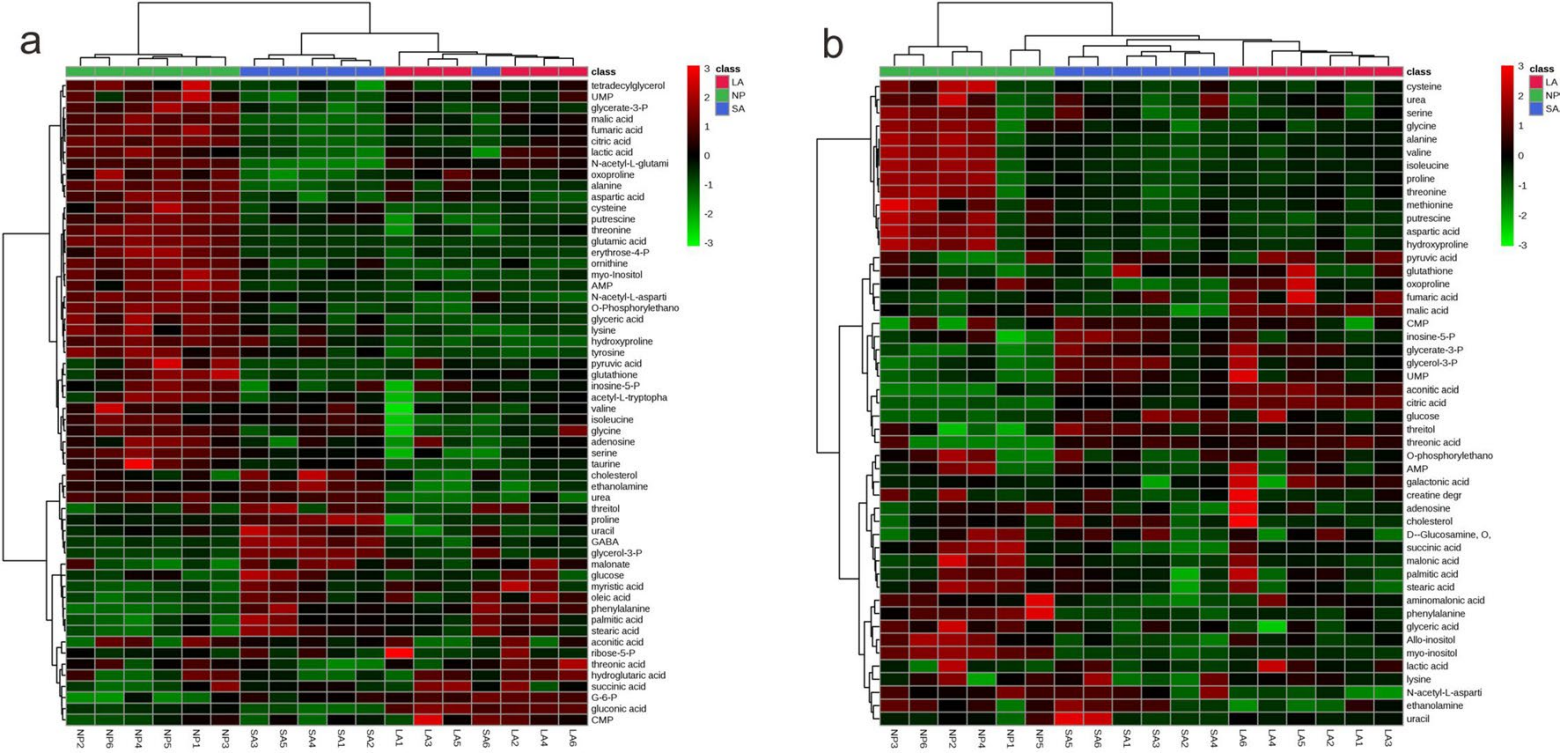
Heat maps of the identified metabolites in MDA-MB-231 (**a**) and MCF-7 (**b**).

There are 58 and 49 metabolites were identified in MDA-MB-231 and MCF-7, respectively. The identified metabolites in MDA-MB-231 and MCF-7 were used to create the heat maps hierarchical clustering (Fig. 3). Figure 3 showed the three groups of MDA-MB-231 were basically discriminated and one of the SA sample was mixed into group LA, while in MCF-7, the groups were clustered well. The groups of SA and LA were similar compare with NP group, while there existed differences.

In order to compare the differences of three groups in MDA-MB-231 and MCF-7, we screen out the significantly changed metabolites. Significantly changed metabolites in all cell lines at different culture conditions were obtained based on the criteria of VIP > 1.0 and *P* < 0.05, which were summarized in Tables 1, 2 and Supplementary Table S1-S2. Under acidosis condition, sixteen metabolites were significantly changed in MDA-MB-231 cells (Table 1), including 11 decreased amino acid metabolites and 5 increased metabolites related to fatty acid metabolism. In MCF-7 cells, eleven metabolites were significantly decreased including amino acids and inositol, and four were significantly increased including glycolytic metabolites, citric acid and inosine 5′-monophosphate (IMP) (Supplementary Table S1). Under lactate acidosis, fifteen significantly metabolites were decreased and gluconic acid, palmitic acid and phenylalanine were increased in MDA-MB-231 (Table 2) when compared to normal condition. In MCF-7, eight significantly decreased metabolites were obtained including amino acids and inositol and five significantly increased metabolites including glycolytic metabolites, TCA metabolites and IMP (Supplementary Table S2). No significant changes in fatty acid were found in MCF-7 under both acidosis and lactic acidosis, while glycerol-3-P, one of glycolytic metabolites, also a skeleton compound of lipid synthesis, was significantly increased in both cell lines.

**Table 1:** Relative concentration of significantly changed metabolites in MDA-MB-231 cultured at pH 6.5 (mean ± SD).

No	Name	Retention time (s)	VIP	*P* value	Fold change (SA/NP)	NP_mean	SA_mean
1	Inositol	1829.80	8.59	2.37E−05	0.69	1.44 ± 0.13E + 08	9.93 ± 0.74E + 07
2	Lactate	419.75	7.87	4.65E−05	0.66	1.13 ± 0.11E + 08	7.46 ± 0.82E + 07
3	Proline	772.96	6.55	5.19E−03	1.50	6.47 ± 0.21E + 07	9.67 ± 2.19E + 07
4	Oxoproline	1109.47	5.55	1.29E−03	0.68	6.60 ± 0.79E + 07	4.48 ± 0.86E + 07
5	Alanine	474.73	3.70	8.44E−08	0.38	1.27 ± 0.07E + 07	4.88 ± 1.23E + 06
6	Threonine	912.18	2.65	8.27E−07	0.51	8.38 ± 0.56E + 06	4.29 ± 0.74E + 06
7	Glutamic acid	1254.96	2.62	6.09E−10	0.16	4.61 ± 0.39E + 06	7.26 ± 1.59E + 05
8	Glycerol-3-P	1441.45	1.91	2.10E−10	2.06	1.94 ± 0.16E + 06	3.99 ± 0.12E + 06
9	Palmitate acid	1781.48	1.56	1.53E−03	1.51	3.30 ± 0.51E + 06	4.99 ± 0.81E + 06
10	Citric acid	1512.52	1.45	1.20E−08	0.50	2.41 ± 0.12E + 06	1.21 ± 0.13E + 06
11	Phenylalanine	1144.82	1.44	3.01E−04	2.35	9.96 ± 1.63E + 05	2.34 ± 0.59E + 06
12	Stearic acid	1993.75	1.30	1.98E−04	1.49	2.22 ± 0.29E + 06	3.30 ± 0.37E + 06
13	Tyrosine	1653.09	1.25	1.08E−03	0.50	2.13 ± 0.44E + 06	1.06 ± 0.38E + 06
14	N-Acetyl-L-Glutamic acid	1464.95	1.08	3.54E−06	0.54	1.49 ± 0.05E + 06	8.06 ± 1.76E + 05
15	Putrescine	1403.13	1.07	5.42E−04	0.58	1.81 ± 0.20E + 06	1.05 ± 0.32E + 06
16	Aspartic acid	1112.58	1.02	4.51E−05	0.12	7.27 ± 1.92E + 05	8.97 ± 2.22E + 04

**Table 2:** Relative concentration of significantly changed metabolites in MDA-MB-231 cultured at pH 6.5 with 10 mmol/L lactate (mean + SD).

No	Name	Retention time (s)	VIP	*P* value	Fold change (LA/NP)	NP_mean	LA_mean
1	Inositol	1829.80	11.55	3.11E−06	0.60	1.44 ± 0.13E + 08	8.65 ± 0.80E + 07
2	Oxoproline	1109.47	3.92	3.57E−02	0.84	6.60 ± 0.79E + 07	5.56 ± 0.68E + 07
3	Alanine	474.73	3.36	4.97E−04	0.58	1.27 ± 0.68E + 07	7.34 ± 2.52E + 06
4	Isoleucine	768.53	3.20	2.50E−03	0.72	1.89 ± 0.17E + 07	1.36 ± 0.27E + 07
5	Glutamic acid	1254.96	3.10	3.69E−10	0.14	4.61 ± 0.39E + 06	6.54 ± 1.19E + 05
6	Threonine	912.18	2.88	6.95E−05	0.55	8.38 ± 0.56E + 06	4.62 ± 1.30E + 06
7	Valine	645.57	2.00	4.86E−02	0.78	1.26 ± 0.20E + 07	9.79 ± 2.30E + 06
8	Taurine	1307.83	1.97	6.78E−03	0.19	2.61 ± 1.31E + 06	4.83 ± 3.69E + 05
9	Tyrosine	1653.09	1.94	7.69E−06	0.23	2.13 ± 0.44E + 06	4.84 ± 1.95E + 05
10	Putrescine	1403.13	1.57	1.77E−05	0.40	1.81 ± 0.20E + 06	7.25 ± 2.85E + 05
11	Phenylalanine	1144.82	1.54	4.52E−05	2.05	9.96 ± 1.63E + 05	2.04 ± 0.34E + 06
12	Palmitic acid	1781.48	1.38	9.13E−03	1.33	3.30 ± 0.51E + 06	4.38 ± 0.64E + 06
13	Citric acid	1512.52	1.34	3.90E−05	0.66	2.41 ± 0.12E + 06	1.60 ± 0.26E + 06
14	Urea	736.71	1.25	2.95E−05	0.28	9.80 ± 1.24E + 05	2.78 ± 2.05E + 05
15	Glycerol-3-P	1946.17	1.22	5.37E−05	0.76	2.75 ± 0.23E + 06	2.08 ± 0.09E + 06
16	Gluconic acid	1682.51	1.20	7.68E−07	1.52	1.17 ± 0.10E + 06	1.78 ± 0.10E + 06
17	Glycine	786.86	1.19	2.31E−02	0.83	4.84 ± 0.16E + 06	4.01 ± 0.74E + 06
18	Lysine	1634.06	1.19	4.30E−05	0.21	7.96 ± 2.07E + 05	1.66 ± 0.87E + 05
19	Serine	873.10	1.09	1.44E−03	0.56	1.36 ± 0.12E + 06	7.65 ± 3.13E + 05

**Figure 4:**
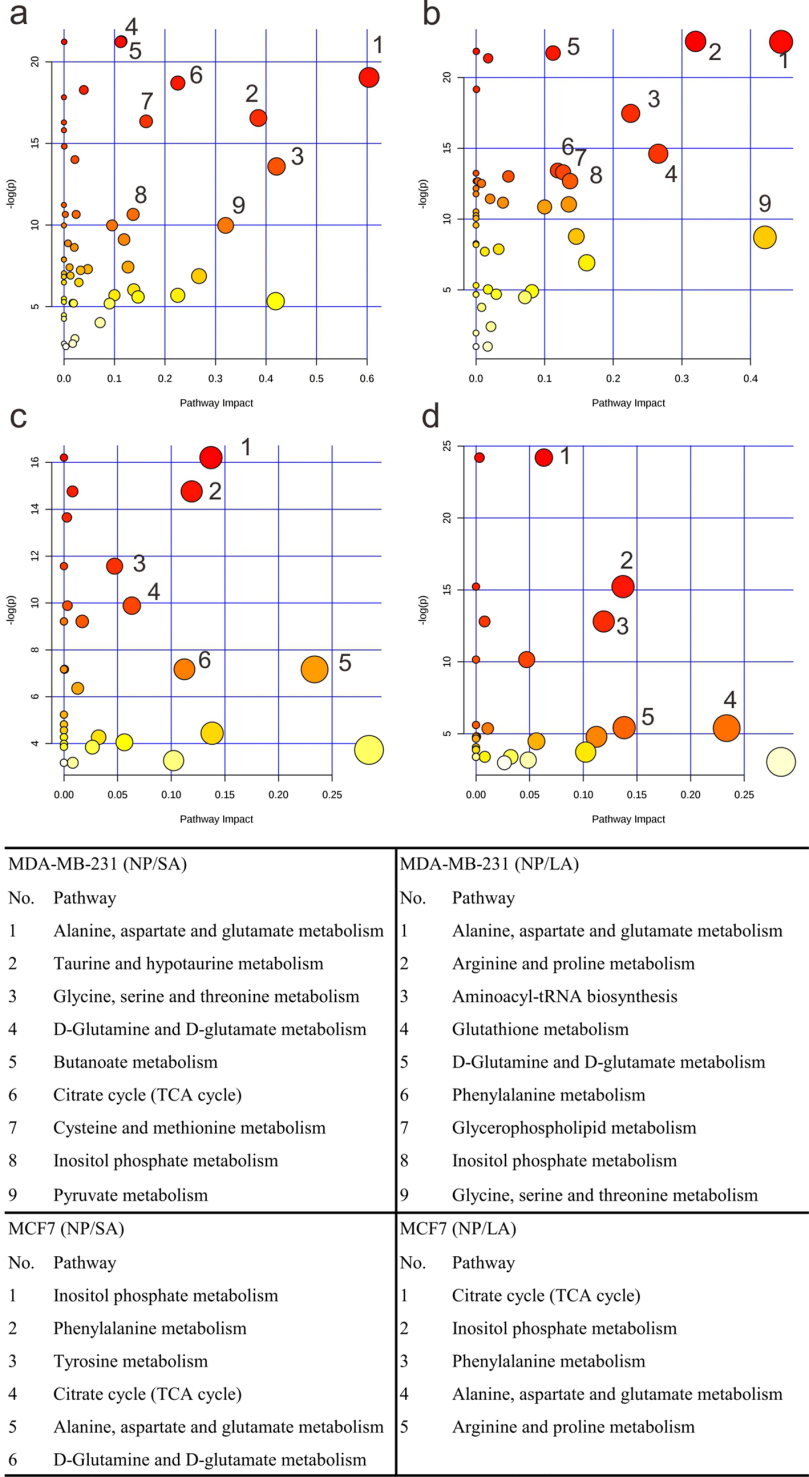
Affected metabolic pathways in MDA-MB-231 (**a**,**b**) and MCF-7 (**c**,**d**) cells under acidosis (**a**,**c**) and lactic acidosis (**b**,**d**).

Identification of affected pathways in acidosis and lactic acidosis was based on enrichment analysis using MetaboAnalyst (Fig. 4). The results showed that three common pathways were affected by acidosis in two cell lines, which include: citrate cycle (TCA cycle), alanine, aspartate and glutamate metabolism, D-glutamine and D-glutamate metabolism. Pathways modulated by lactic acidosis in two cell lines include inositol phosphate metabolism, phenylalanine metabolism, alanine, aspartate and glutamate metabolism, arginine and proline metabolism. The affected pathways in MDA-MB-231 was more than that in MCF-7, which indicated that MDA-MB-231 was more active when faced with the external disturbance.

### Isotope-assisted metabolomic analysis of cell metabolism under acidosis and lactic acidosis

GC–MS and NMR studies were carried out on the cell extracts of MDA-MB-231 and MCF-7 cultured in media with different labeled substrates (1:1 mixture of U-^13^C_6_-glucose and 1-^13^C-glucose and 1,2-^13^C_2_-glucose) under acidosis and lactic acidosis conditions. The labeled substrates can produce distinct labeling patterns of intermediates, making an outline of metabolic pathways activity in the given cell lines.

As shown in Supplementary Fig. S3, the scheme shows labeled patterns of pyruvate from glucose. When the oxidative branch of PPP is operative, glucose would lose the first carbon as CO_2_ and U-^13^C_6_-glucose and 1-^13^C-glucose converted to U-^13^C_3_-pyruvate (M3) and unlabeled pyruvate (M0), respectively. When 1-^13^C-glucose metabolized via glycolysis pathway, unlabeled pyruvate and 3-^13^C-pyruvate (M1) would be generated. Therefore, the relative contents of M1 pyruvate were used to estimate the flux of pyruvate from PPP. The lower the relative content is, the more active the PPP pathway is. In MDA-MB-231 cells, significantly decreased M1 pyruvate content indicated that PPP was enhanced in lactic acidosis group, while M1 pyruvate only showed slight change in MCF-7 cells.

**Figure 5:**
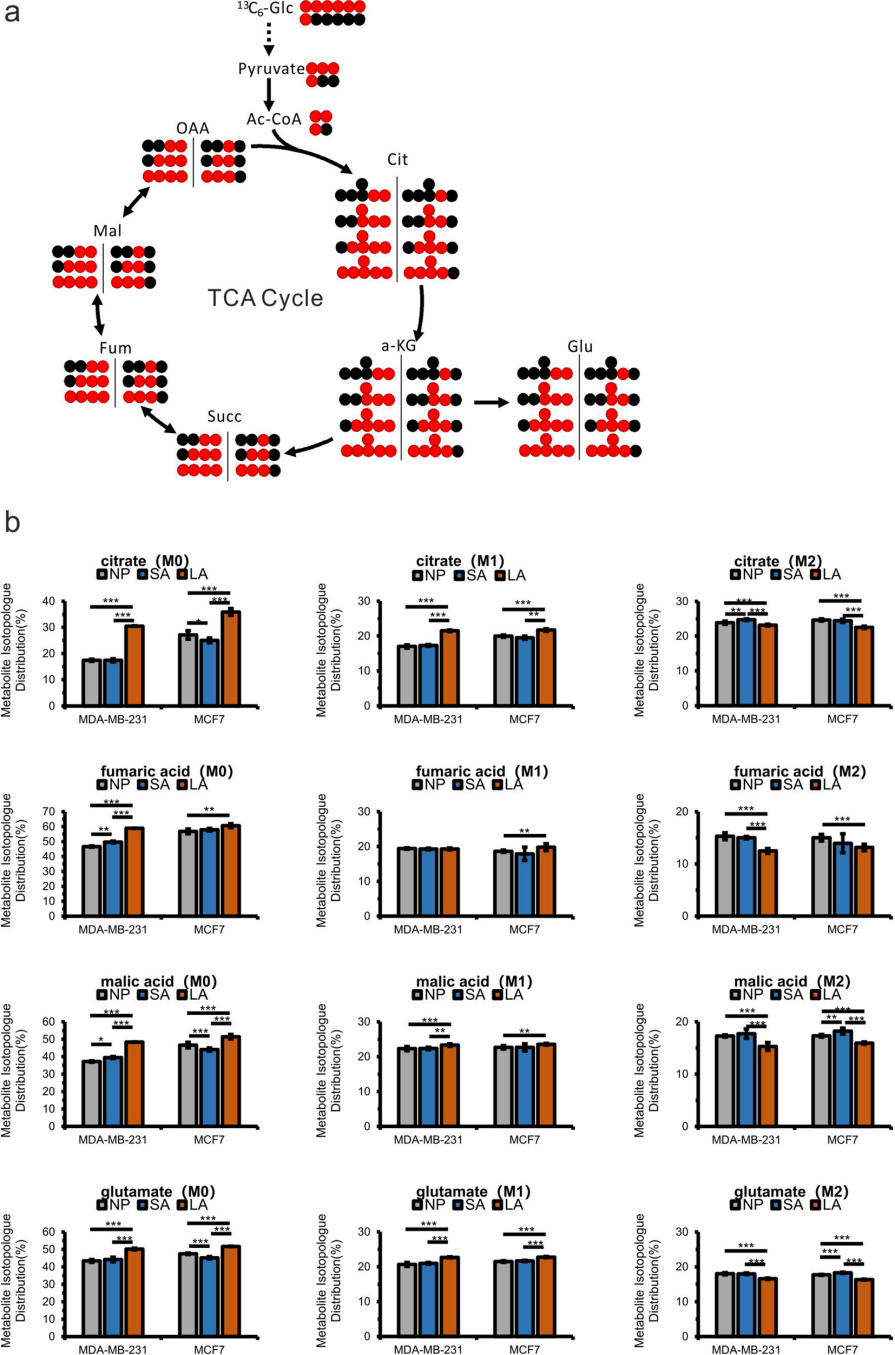
Changes in TCA cycle metabolism. (**a**) The labeling scheme showing ^13^C atom derived from U-^13^C_6_-glucose and 1-^13^C-glucose from glucose (red dots). The black dots represent ^12^C atoms. (**b**) Comparisons of mass isotopologue distributions (MIDs) of citrate, fumaric acid, malic acid and glutamate of MDA-MB-231 and MCF-7 cells.

As shown in Fig. 5a, the scheme shows the fate of carbons originated from glucose. U-^13^C_6_-glucose and 1-^13^C-glucose can be metabolized to M3 pyruvate and M1 pyruvate, which can be further converted into M2 acetyl-CoA and M1 acetyl-CoA which participates in the TCA. The mass isotopologue distribution (MID) of fumaric acid and malic acid can be used to indicate the conversion rate of glucose into TCA cycle. In both cell lines, the significantly higher M0 and lower M2 components of citrate, fumaric acid and malic acid of all four metabolites in lactic acidosis group suggested the contribution of other sources such as lactate and a decreased conversion rate of glucose into TCA cycle (Fig. 5b). Glucose can be converted into α-ketoglutarate and glutamate (Fig. 5a). The percentage of M2 glutamate (Fig. 5b) was lower in LA, indicating a decreased conversion flux from glucose to glutamate under lactic acidosis condition. In Fig. 5b, M1 metabolites were increased in LA, while M1 pyruvate was decreased in LA (Supplementary Fig. S3), which indicated that pyruvate in LA converted into other pathways decreased such as lactate (Fig. 2). For MDA-MB-231 cells, only slight changes of these metabolites were observed in acidosis condition whereas for MCF-7 cells, M2 malic acids were increased (Fig. 5b), which indicated that TCA pathway was not sensitive to acidic environment in MDA-MB-231 an increased conversion rate of glucose into TCA cycle in MCF-7.

Pyruvate enters TCA cycle not only via pyruvate decarboxylation but also via carboxylation. Pyruvate can be converted to oxaloacetate under the catalysis of pyruvate carboxylase (PC). M3 pyruvate was converted to M3 oxaloacetate and produced M3–M5 citrate (Supplementary Fig. S4). As shown in Supplementary Fig. S4, significantly decreased M3–M5 citrate in lactic acidosis group indicated that pyruvate carboxylation was decreased in both cell lines, while in acidosis group increased M3–M5 citrate in MCF-7 showed that pyruvate carboxylation was increased slightly.

^13^C NMR experiments of MDA-MB-231 cell extracts cultured with a 1:1 mixture of U-^13^C_6_-glucose and 1-^13^C-glucose were also carried out, and the spectral assignments are shown in Supplementary Fig. S5. We can also see that the content of unlabeled acetate was increased in SA and LA (Supplementary Fig. S6). Significantly increased unlabeled acetate in lactic acidosis group may be caused by other carbon sources such as lactate. In MCF-7 the labeled acetate was significantly decreased in acid media, while this change is not obvious in MDA-MB-231. Studies have showed that acetate was released more in acidosis via histone deacetylation to regulate intracellular pH^12^. The unlabeled acetate originated from histone deacetylation, the metabolism of other carbon sources and the undialyzed serum. ATP-citrate lyase (ACLY) could make citrate cleavage, with subsequent metabolites of OAA and acetyl-CoA^42^. Acetyl-CoA can be further converted to acetate. The level of labeled acetate illustrated that acidosis, especially lactic acidosis decreased glucose-to-acetate metabolic switch in MCF-7, while MDA-MB-231 was not sensitive to this stimulation.

**Figure 6:**
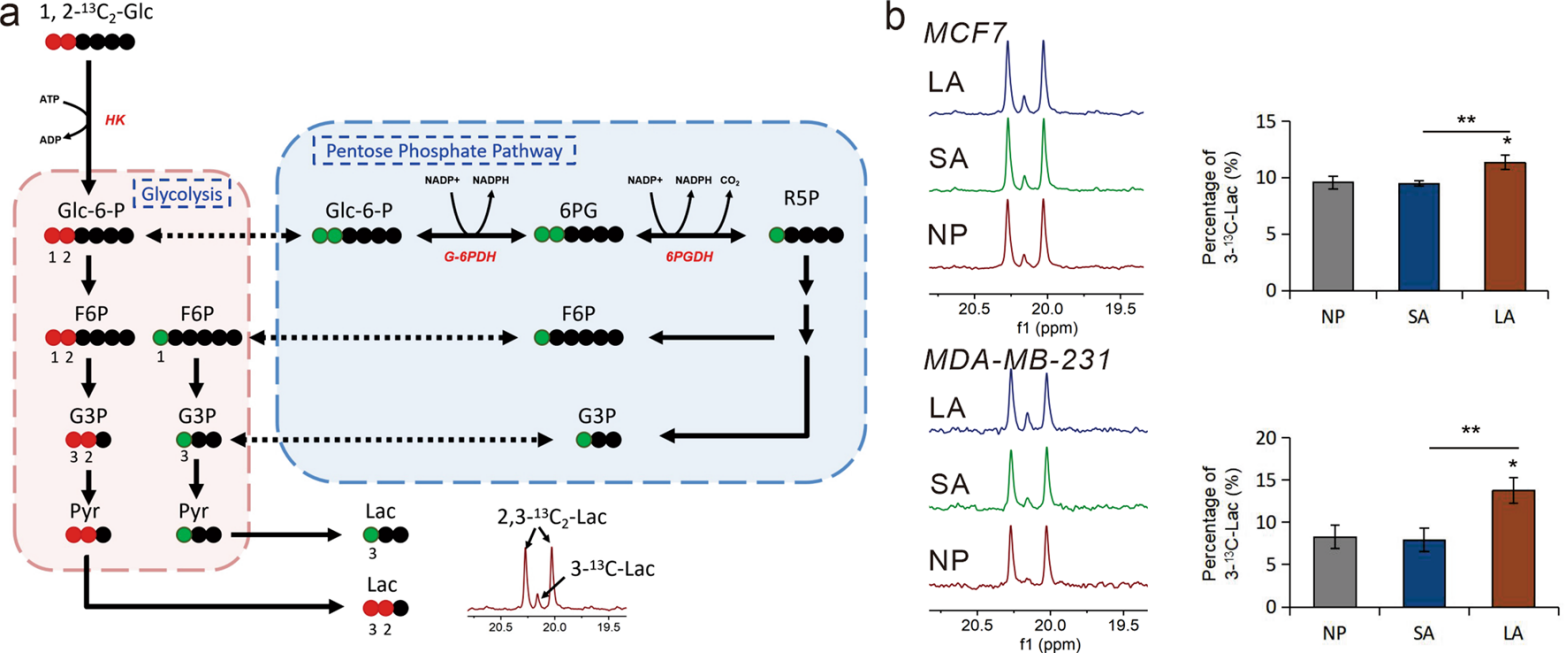
Metabolism of 1,2-^13^C_2_ glucose through glycolysis and pentose phosphate pathway (PPP) with the labeling pattern of lactate. (**a**) The scheme shows the fate of individual carbons through glycolysis (red dots, ^13^C) and PPP (green dots, ^13^C), labeling in Glc-6-P, F6P, G3P, Pyr and Lac. Black dots represent ^12^C atoms. (**b**) ^13^C-NMR spectra (left) and the corresponding histogram (right) from MDA-MB-231 and MCF-7 media after cultured in NP, SA and LA. 3-^13^C-Lac (%) = [3-^13^C-Lac] / ([3-^13^C-Lac] + [2,3-^13^C_2_-Lac] ).

In order to evaluate the effects of acidosis and lactic acidosis on PPP, MCF7 and MDA-MB-231 cells were cultured in media supplemented with 1,2-^13^C_2_-glucose for 48 h, and the ^13^C NMR data of media were collected. The 1,2-^13^C_2_-glucose is metabolized into 2,3-^13^C_2_-pyruvate through glycolysis and 3-^13^C-pyruvate through PPP, followed by the production of 2,3-^13^C_2_-lactate with a doublet and 3-^13^C-lactate with a singlet (Fig. 6a). The relative ^13^C fractional enrichments of excreted 3-^13^C-lactate were evaluated by the integrating signals relative to labeled lactate C3. As shown in Fig. 6b, increased percentage of 3-^13^C-lactate verified that PPP in both cell lines are metabolically more active in lactic acidosis.

## Discussion

It was found that viability of different breast cancer cell lines under acidic milieu was discrepant. The cells viability was decreased under acidosis and lactate could reduce the acidosis damage to cells. Utilization and excretion of lactic acid depend on the concentration of lactic acid intracellular and extracellular and the expression of MCTs. In MDA-MB-231 and MDA-MB-468, both being basal-like and triple negative cell lines, expression of MCTs was different. MCT1 is expressed in MDA-MB-468 but could barely be detected in MDA-MB-231^43,44^. Several studies have been published on the acidosis and its effects on phenotype in tumorigenesis, however, little is known about the cellular metabolic response to acidosis and lactate acidosis in most solid tumors. Here we tested the relative content of glucose and lactate in the medium, which are the main metabolites of glycolysis, and employed untargeted and stable isotope-assisted metabolomics to investigate metabolites and pathways changes. In this study, decreased glucose and lactate in the medium reflected decreased glycolysis in acidosis and the results of ^13^C NMR and glycolysis stress indicated that lactate acidosis could increased glycolysis. TCA cycle plays a key role in generating energy and providing precursors of certain amino acid as well as reducing agent NADH used in many other biochemical reactions. With the previous studies, our present data showed that levels of TCA cycle-related metabolites citric acid, fumaric acid and malic acid were decreased in low pH in MDA-MB-231 (Supplementary Fig. S7). There was a statistically significant difference (*P* < 0.05) in the content of malonate between SA and NP, while between LA and NP, the malonate occurs at elevated concentration with no significant difference. Hence, a slight elevation of TCA cycle metabolites in LA was observed in comparison with SA group. In MCF-7 only slight changes with no significant differences were observed (Supplementary Fig. S8). Malonate that is analogue of succinate, a succinate dehydrogenase inhibitor of TCA cycle, which increases in low pH, could be an influence factor of making TCA decrease. Citric acid usually used to support de novo synthesis of fatty acid, other TCA cycle metabolites are used for biosynthesis of various macromolecules.

ATP-citrate lyase (ACLY) could make citrate cleavage, with subsequent metabolites of OAA and acetyl-CoA^42^. The obtained acetyl-CoA is a key metabolite, which connect glycolysis, TCA and fatty acid metabolism. Acetyl-CoA can also make modification for histone acetylation, which is of great importance for formulation and development of tumors. Acetyl-CoA furthermore converted to acetate to be detected. Studies have showed that acetate was released more in acidosis via histone deacetylation to regulate intracellular pH^12^. From the results, on one hand, the unlabeled acetate originated from histone deacetylation, on the other hand, it comes from metabolism of other carbon sources. The level of labeled acetate illustrated that acidosis, especially lactate acidosis decreased glucose-to-acetate metabolic switch in MCF-7, which may suggest that acidosis and lactic acidosis could decrease ACLY expression, while MDA-MB-231 was not sensitive to this stimulation.

Pentose phosphate pathway is another important metabolic pathway for cell survival. PPP can produce carbon skeleton of ribose or derivatives, such as ATP, CoA, NAD+, FAD, RNA or DNA, which are crucial constituent part of biological molecules. For another, PPP is the main source of NADPH. NADH is mainly used to provide ATP, while unlike NADH, NADPH can provide hydrogen ions in reducing biosynthesis^45,46^. Therefore, reduced NADPH can favor fatty acid and steroid biosynthesis^47^. In our study, PPP was increased in lactic acidosis with the help of stable isotope-assisted metabolomics in MDA-MB-231 and MCF-7. The cell viability enhanced in lactic acidosis compared to that in acidosis can be accounted for the increased PPP flux.

Lipids are of importance to the membrane formation, energy storage and signal transduction. Dysregulation of de novo lipogenesis is increasingly recognized as a hall mark of cancer. Increased lipid synthesis fuels cancer energy and contributes to production of membranes for cell proliferation under metabolic stress, such as hypoxia and acidosis^48,49^. Studies have shown that fatty acid synthase (FAS) overexpression promoted apoptosis and proliferation in cancer cells^50^. We can observe that the fatty acids (stearic acid, palmitic acid, oleic acid, myristic acid) and cholesterol increased in low pH with significant differences in MDA-MB-231 cells, while that of MCF-7 showed minor changes (Supplementary Figure S7, S8). Some studies show that acidosis drives FA metabolism reprogramming in cancer cells, which can be a reason that MDA-MB-231 survived in acidosis according to the viability tests. This metabolic disturbance makes cancer cells overcome the metabolic stress^51^.

In conclusion, we compared the metabolic changes of two acidosis modes on MDA-MB-231 cells and MCF-7 cells in our study. The activity of breast cancer cells was reduced after exposure to acute acid stimulation, while exposure to lactic acidosis was associated with an enhancement of cell viability compared to acidosis. Acidosis decreases the glycolysis as revealed by downregulated lactic acid in media, while lactic acidosis could promote glycolysis as we observed the elevated ECAR in lactic acidosis. The M1 pyruvate and labeling pattern of lactate C3 indicated that PPP flux increased with the lactic acidosis stimulating. Both acidic irritations can promote fatty acid synthesis in MDA-MB-231. These results indicated that cell metabolic changes existed in acidosis, while both the acidosis caused different metabolic reprogramming. Addition of lactate can rescue the low viability of cancer cells in acute acid environment. All these metabolic changes in cancer cells caused by lactate in acidosis can provide valuable information, which could help to get known about the underlying metabolism of cancer cells.

## Materials and methods

### Cell culture and extraction

MCF-7, MDA-MB-468, MDA-MB-231, and SkBr3 cells were obtained from Hunan Fenghui Biotechnology Co., Ltd. All cells were maintained in Dulbecco’s Modified Eagle Medium (DMEM, Gibco) containing 10% Fetal Bovine Serum (FBS, Gibco) and 1% penicillin–streptomycin (HyClone). Cells were subcultured to 10 cm plates by rinsing with PBS (Gibco) and detaching with 0.5% trypsin–EDTA (Sigma). Cells were grown in an incubator (SANYO, MCO-20AIC) containing 5% CO_2_ and ambient oxygen at 37 °C. Simple acidosis (SA) was generated via media pH adjusted to pH 6.5 using HCl, while lactic acidosis (LA) was generated via the addition of 10 mmol/L sodium lactate (Sigma), then media pH adjusted to pH 6.5 using HCl (Lactate, with a relatively weak dissociation, has an limited impact on the buffer capacity). The media contain 10 mmol/L glucose. All groups were cultured for 48 h.

For ^13^C-glucose labeling experiments, cells were plated in high glucose DMEM overnight in 10 cm or 15 cm dishes, and then rinsed with PBS and switched to media containing 5 mmol/L U-^13^C_6_-glucose and 5 mmol/L 1-^13^C-glucose or 10 mmol/L 1,2-^13^C_2_-glucose (CIL, Cambridge Isotope Laboratories) under control, acidosis or lactic acidosis condition and cultured for 48 h.

Culture media and cell pellets were collected after cells were rinsed twice in 1 × PBS, quenched and scraped in 1 mL of 80% methanol/H_2_O. The cell suspension was ultrasonicated in ice bath twice with 20 min each time and vortexed for 30 s, and then centrifuged at 13,000×*g* for 20 min at 4 °C. The supernatant was collected into 2 mL vial and added with 20 µL of 0.2 mg/mL ribitol (Sigma-Aldrich) as an internal standard. The procedures for derivatization of the supernatant were performed as previously reported^7^. For GC–MS, six replicates of each sample were performed and for NMR, three replicates of each sample were performed in the subsequent detection.

### Cell treatment and cell viability assay

Cell viability of cancer cell lines (MCF-7, MDA-MB-231, MDA-MB-468 and SkBr3) was assessed in acidosis and lactic acidosis by colorimetric assay with Cell Counting Kit-8 (CCK-8 solution, Beijing Applygen Technologies Inc.). Cancer cells were plated in 96 well culture plates at a concentration of 5 × 10^3^ cells per well and allowed to attach for 12 h. Cells were treated with acidosis medium (pH 6.5) and lactic acidosis medium (10 mmol/L lactate, pH 6.5) with a volume of 200 μL and incubated for 48 h. After the addition of the CCK-8 solution (10 μL/well), the reaction was incubated for 2 h and a reading of optical densities (OD) was taken at 450 nm using an ELISA reader (Biotek, Elx808, USA). The viability (%) was calculated based on the following formula: Viability (%) = (OD_tr_–OD_bl_) / (OD_ntr_–OD_bl_). (OD_tr_ is the mean OD of the treated cultures, OD_ntr_ is the mean OD of the non-treated cultures and OD_bl_ is mean OD of the blank-wells).

### Extracellular acidification

To measure extracellular acidification rate (ECAR), 20,000 cells from each cell line were seeded into each well of a XF24 microplate in a total of 100 μL for 4 h. Cells were subsequently treated with pH 7.4 and pH 6.5 medium (with or without 10 mmol/L lactate) for 48 h. ECAR were measured using a Seahorse Bioscience Extracellular Flux Analyzer (XF24). The data were analyzed using the Seahorse XF software.

### Sample derivatization and GC–MS analysis

The sample derivatization method is the same as previously described^7^. The extracts were added with 40 µL pyridine solution of 20 mg/mL methoxyamine hydrochloride (Sigma-Aldrich), vortexed for 30 s and incubated at 30 °C and 130 rpm for 90 min in a gas bath shaker, followed by addition of 40 µL MSTFA (Sigma-Aldrich) with 1% chlorotrimethylsilane (TMCS, Sigma-Aldrich) and incubated at 37 °C for further 30 min. The derivatized samples were stored at 4 °C before detection.

The samples were analyzed with an Agilent 6890 GC system coupled with a Pegasus 4D time-of-flight mass spectrometer (Leco Corporation, St Joseph, MI, USA). The column was DB-5 MS (30 m × 250 µm i.d., 0.25 µm, Agilent J&W Scientific, Folsom, CA, USA), with an oven program set at 70 °C for 1 min, increased to 280 °C with a ramp rate of 5 °C/min holding for 15 min. Samples (1 μL) were injected with no split. The temperature of the inlet, interface and ion source was set as 250, 250, 220 °C, respectively. The solvent acquisition delay was 300 s. The mass spectrometer was operated in full scan mode (*m/z* 50–800) with an acquisition rate of 10 spectra/s. Electron impact ionization was set at 70 eV^7,52^.

### ^1^H and ^13^C NMR parameters

^13^C NMR experiments were performed at 298 K using a Bruker AVANCE 950 MHz NMR spectrometer equipped with Cryo probes. ^13^C spectra were acquired with a relaxation delay of 3 s, 5120 scans, and 32 k data points. ^1^H experiments were performed at 298 K using a Varian UNITYINOVA 600 MHz spectrometer. ^1^H NMR spectra of cell media were acquired using a standard PRESAT pulse sequence with a relaxation delay of 2 s, 32 scans, 32 k data points, and a spectral width of 7800 Hz. Spectra were manually phased and baseline-corrected.

For cell extracts, the samples were dissolved in 400 μL D_2_O and 5 μL TSP D_2_O solution (1 mg/mL) for ^13^C experiments, and for culture media, 500 μL of the media was mixed with 80 μL TSP D_2_O solution (1 mg/mL) for experiments.

### Data processing and statistical analyses

Peak picking and alignment of the acquired GC–MS data were conducted by the Statistical Compare feature of ChromaTOF software (Version 4.5, Leco Corp.). Peaks with a signal-to-noise ratio (S/N) greater than 50 were considered and normalized to the intensity of ribitol. Metabolites were identified according to NIST library and the library built with various metabolites standards in our laboratory. The processed data were imported into SIMCA 13.0 software (Umetrics) for multivariate pattern recognition analysis. Principal Components Analysis (PCA) and Projection to Latent Structures Discriminant Analysis (PLS-DA) were applied to compare the chromatograms of samples treated with Pareto scaling (Par)^7^. Pathway analysis on the modulated metabolites was performed using Metaboanalyst 4.0 tool^53^.

For NMR spectra, peaks were normalized to the intensity of TSP. Metabolites were identified according to the library built in our laboratory.

For all other graphs, samples sizes are indicated in the figure legends; *P* values were derived by two-tailed t test. *P* values are indicated (*P ≤ 0.05, **P ≤ 0.01, and ***P ≤ 0.001) and error bars are standard errors of the mean.

## Supplementary Information

Supplementary Information
