# Supplementary material for: Differential metabolic responses in breast cancer cell lines to acidosis and lactic acidosis revealed by stable isotope assisted metabolomics
Source: Sci Rep. 2020 Dec 15;10:21967. doi: 10.1038/s41598-020-78955-2 (PMC7738541; doi:10.1038/s41598-020-78955-2)
Supplement: Supplementary file 1 — Supplementary Information [file 41598_2020_78955_MOESM1_ESM.docx]

# Differential metabolic responses in breast cancer cell lines to acidosis and lactic acidosis revealed by stable isotope assisted metabolomics

Jiayue Gao^1^, Zhiying Guo^1,2^, Jianhua Cheng^1^, Bo Sun^1^, Jie Yang^1^, Haijing Li^1^, Shengming Wu^1^, Fangting Dong^1,*^, Xianzhong Yan^1,*^

^1^National Center of Biomedical Analysis, Beijing 100039, China

^2^Hepatal-Biliary-Pancreatic Center, Beijing Tsinghua Chang Gung Hospital, Beijing102218, China

**The Corresponding Authors**

**Xianzhong Yan**

National Center of Biomedical Analysis

No. 27 Taiping Road, Beijing, 100039, China.

E-mail: yanxz@proteomics.cn (X.Yan).

**Fangting Dong**

National Center of Biomedical Analysis

No. 27 Taiping Road, Beijing, 100039, China.

E-mail: dft@proteomics.cn (F.Dong).


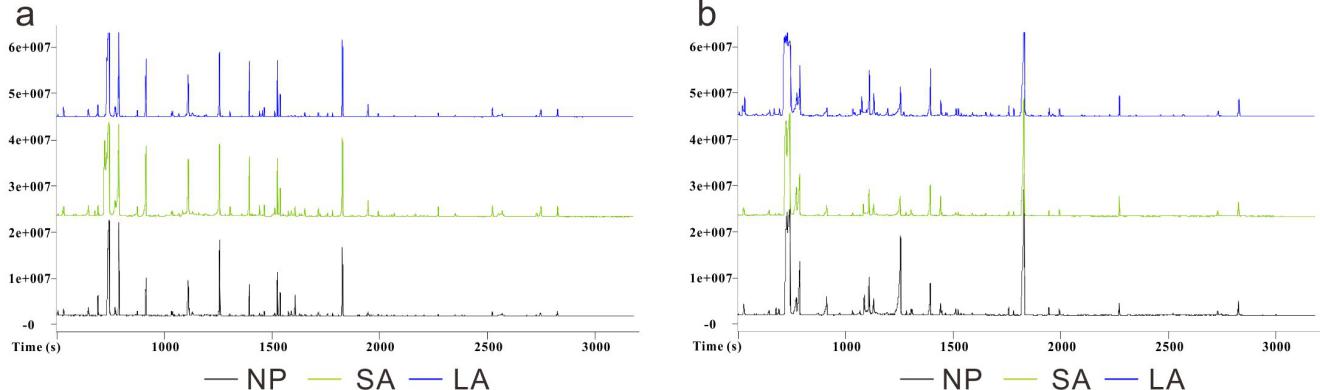


**Figure S1.** Total ions chromatograms (TIC) of intracellular extracts of MDA-MB-231 (a) and MCF7 (b) cell lines in NP, SA and LA.


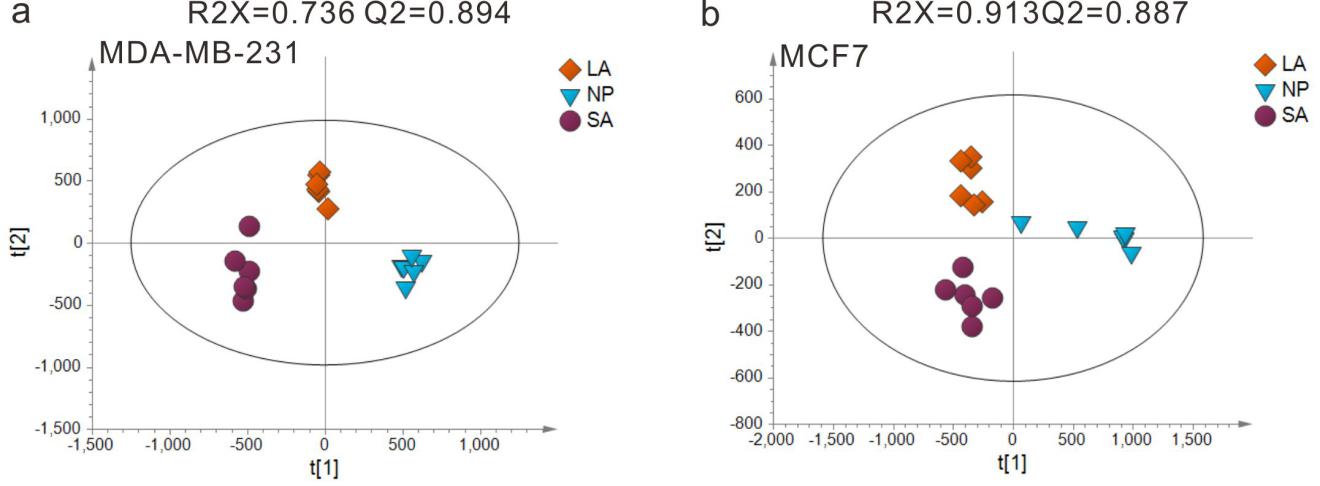


**Figure S2.** PLS-DA score plot for NP, SA and LA of MDA-MB-231 (a) and MCF7 (b) cell lines. The parameters, R2X and Q2, were analysed to ensure the quality of the model and avoid the risk of over-fitting.


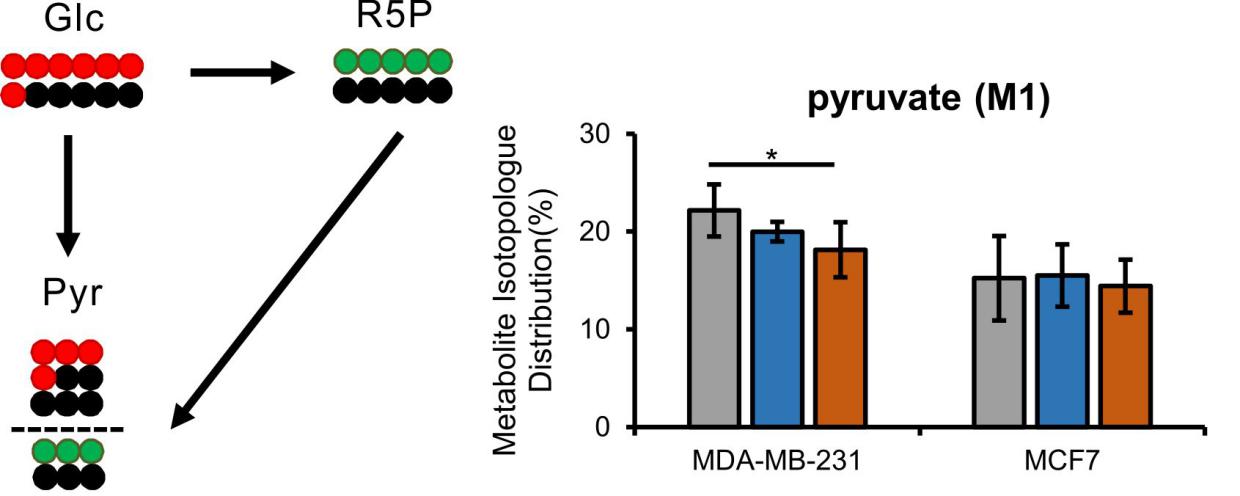


**Figure S3.** Schema showed pyruvate metabolized from PPP and glycolysis. Red filled circles and green filled circles represent ^13^C atom via glycolysis and PPP respectively, black filled circles represent ^12^C atom. The histogram showed the isotopologue distribution of M1 pyruvate ( **P*<0.05).


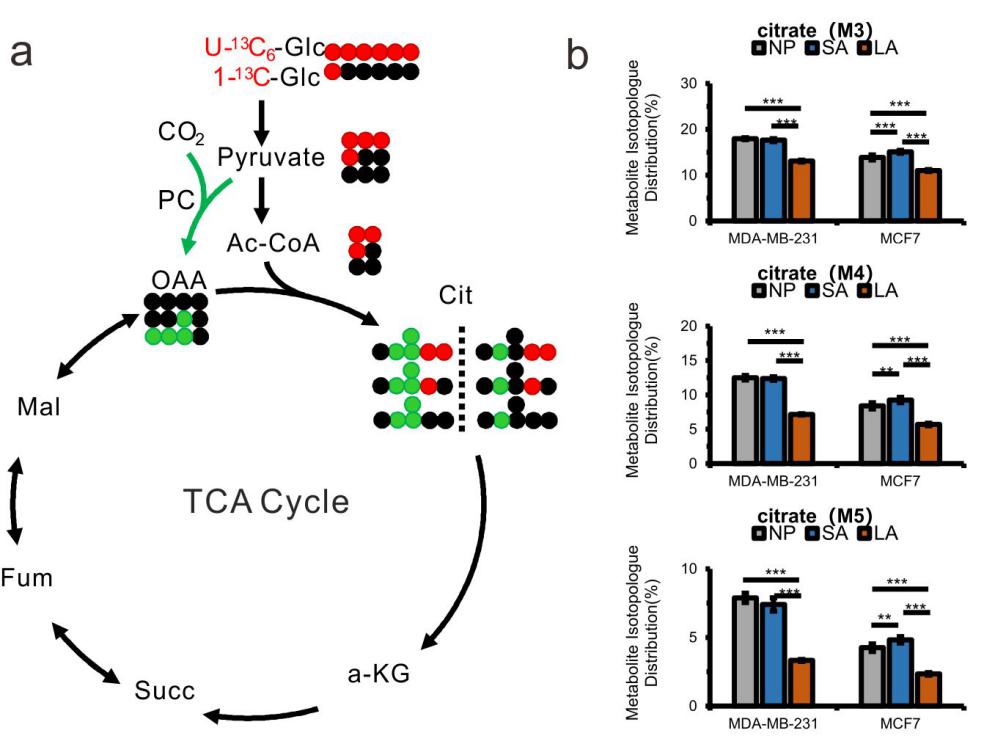


**Figure S4.** Isotopic citrate (M3, M4 and M5) derived from U-^13^C_6_-glucose and 1-^13^C-glucose. Schema showing ^13^C atom transitions via pyruvate carboxylation (a). Red filled circles and green filled circles represent ^13^C atoms via pyruvate decarboxylation and and pyruvate carboxylation, respectively. Black filled circles represent ^12^C atom. Comparisons of mass isotopologue distributions (MIDs) of citrate derived from U-^13^C_6_-glucose and 1-^13^C-glucose of MDA-MB-231 and MCF7 cells (b).


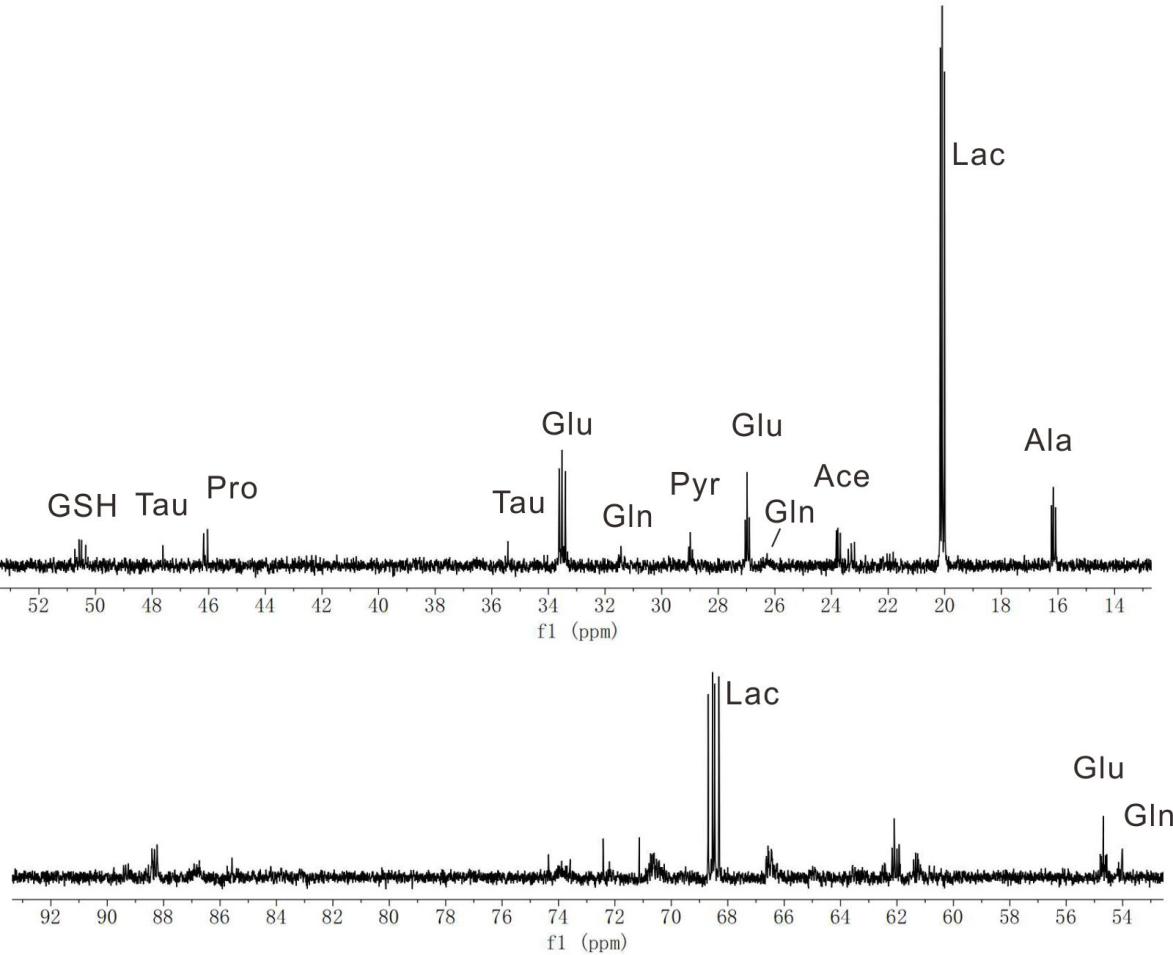


**Figure S5.** ^13^C NMR spectra of MDA-MB-231 cell extracts with the spectral assignments in the LA group.


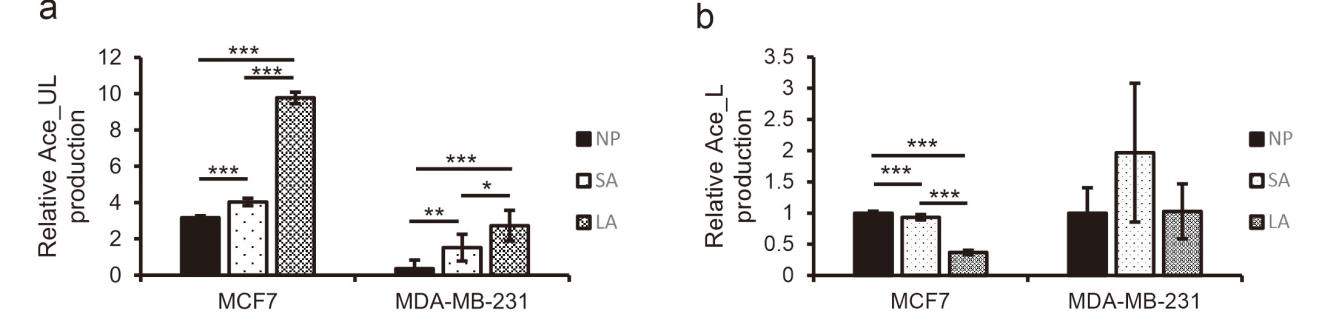


**Figure S6.** Unlabeled acetate and labeled acetate excreted in MDA-MB-231 and MCF7 over 48h were quantified from ^1^H NMR and normalized to the TSP content.


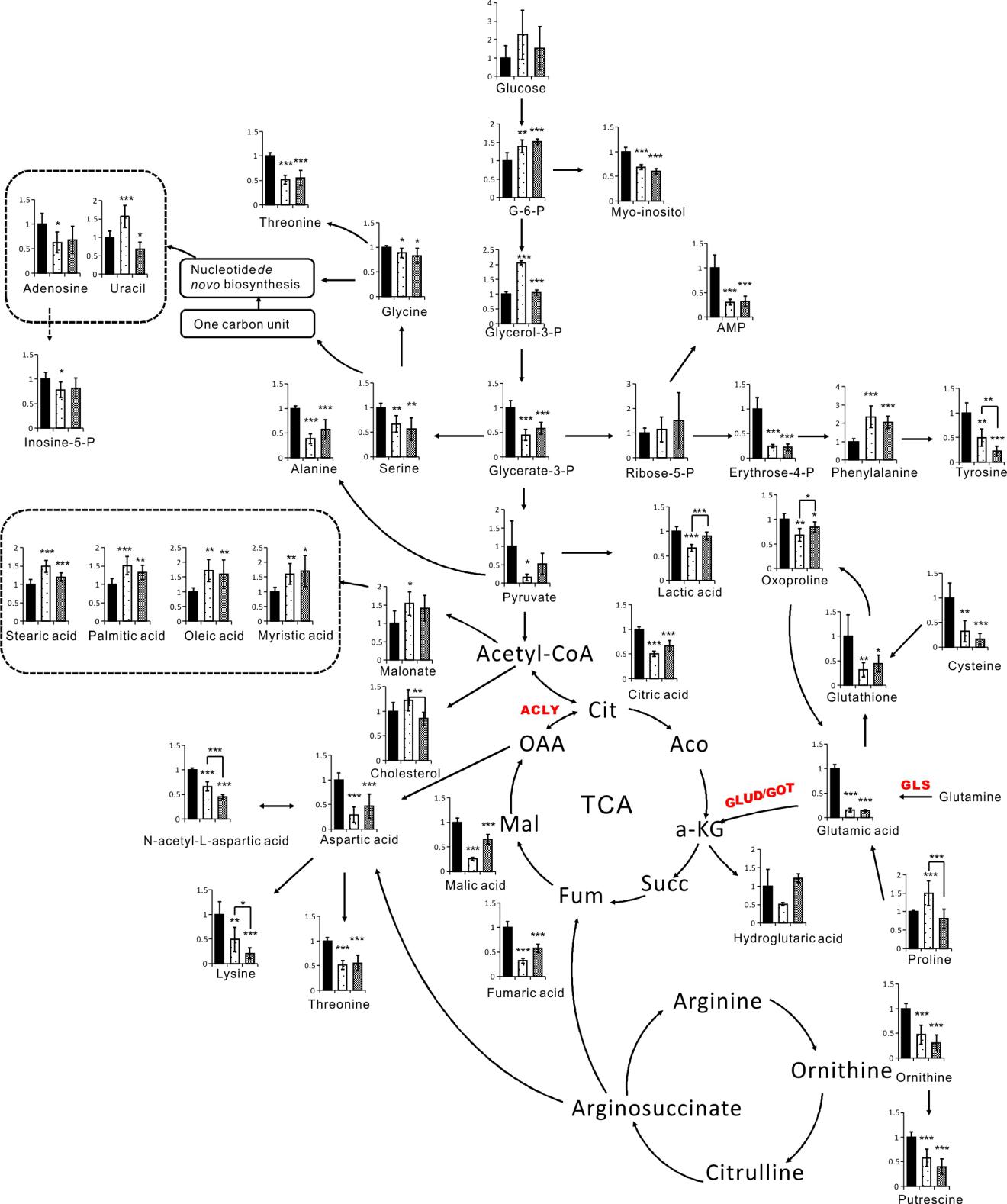


**Figure S7.** Metabolic pathways of MDA-MB-231 cells cultured under acidic media (**P*<0.05, ***P*<0.01, ****P*<0.001). represents for NP, represents for SA and represents for LA.


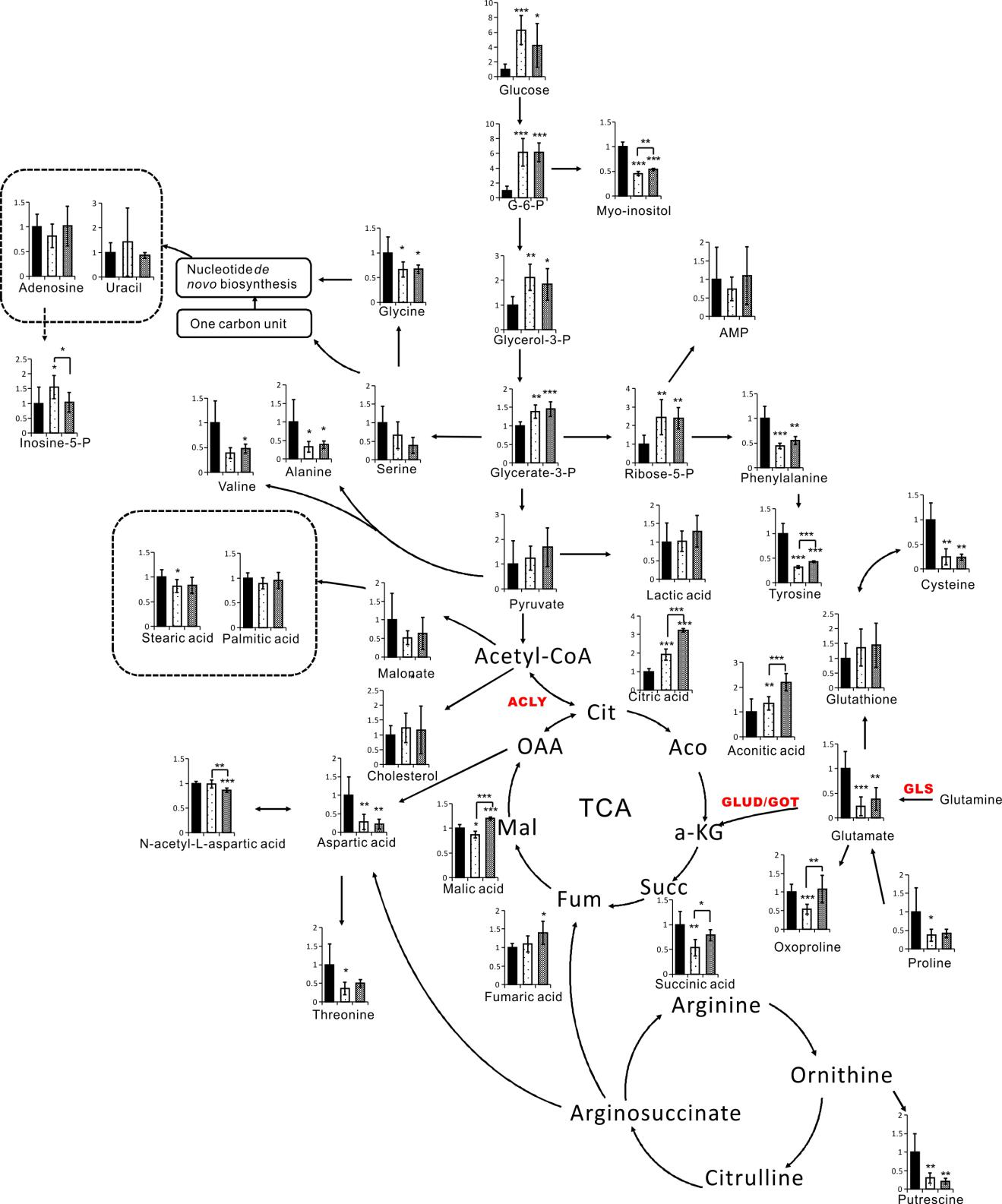


**Figure S8.** Metabolic pathways of MCF7 cells cultured under acidic media (**P*<0.05, ***P*<0.01, ****P*<0.001). represents for NP, represents for SA and represents for LA.

**Table S1.** Relative concentration of significantly changed metabolites of MCF7 under SA vs NP (Mean+SD)

| No. | Name | Retention Time (s) | VIP | *P* Value | FC  (SA/NP) | NP_mean | SA_mean |
| --- | --- | --- | --- | --- | --- | --- | --- |
| 1 | Threonine | 902.36 | 4.53 | 2.26E-02 | 0.36 | 3.12±1.74E+07 | 1.12±0.52E+07 |
| 2 | Glycerol-3-P | 1431.28 | 4.02 | 1.27E-03 | 2.12 | 1.12±0.38E+07 | 2.38±0.59E+07 |
| 3 | Oxoproline | 1097.15 | 3.06 | 9.26E-04 | 0.54 | 1.51±0.31E+07 | 8.11±2.00E+06 |
| 4 | Glutamic acid | 1244.03 | 3.03 | 7.68E-04 | 0.24 | 1.02±0.35E+07 | 2.42±1.91E+06 |
| 5 | Glycine | 776.12 | 2.97 | 4.15E-02 | 0.66 | 2.56±0.81E+07 | 1.69±0.40E+07 |
| 6 | Proline | 761.75 | 2.84 | 4.38E-02 | 0.37 | 1.28±0.83E+07 | 4.78±2.07E+06 |
| 7 | Tyrosine | 1578.13 | 2.56 | 9.41E-06 | 0.32 | 7.32±1.47E+06 | 2.36±0.19E+06 |
| 8 | Isoleucine | 757.67 | 2.13 | 1.28E-02 | 0.35 | 6.52±3.39E+06 | 2.27±0.61E+06 |
| 9 | Valine | 634.73 | 2.07 | 8.07E-03 | 0.39 | 6.47±2.85E+06 | 2.52±0.71E+06 |
| 10 | Phenylalanine | 1133.74 | 1.81 | 2.39E-04 | 0.44 | 4.30±1.03E+06 | 1.89±0.23E+06 |
| 11 | Citric acid | 1502.22 | 1.77 | 5.08E-05 | 1.92 | 2.50±0.37E+06 | 4.79±0.74E+06 |
| 12 | IMP | 2557.67 | 1.48 | 3.95E-04 | 2.60 | 9.90±5.61E+05 | 2.58±0.49E+06 |
| 13 | Myo-inositol | 1814.56 | 1.28 | 9.15E-08 | 0.46 | 2.14±0.19E+06 | 9.76±0.89E+05 |
| 14 | Glucose | 1595.54 | 1.21 | 9.96E-05 | 6.30 | 2.16±1.57E+05 | 1.36±0.42E+06 |
| 15 | Alanine | 964.13 | 1.01 | 1.40E-02 | 0.58 | 2.34±0.72E+06 | 1.36±0.35E+06 |

**Table S2.** Relative concentration of significantly changed metabolites of MCF7 under LA vs NP (Mean+SD)

| No. | Name | Retention Time (s) | VIP | *P* Value | FC  (LA/NP) | NP_mean | LA_mean |
| --- | --- | --- | --- | --- | --- | --- | --- |
| 1 | Glutamic acid | 1120.53 | 4.19 | 8.33E-03 | 0.71 | 5.52±0.94E+07 | 3.91±0.76E+07 |
| 2 | Glycerol-3-P | 1431.28 | 3.18 | 1.71E-02 | 1.84 | 1.12±0.38E+07 | 2.06±0.72E+07 |
| 3 | Glycine | 776.12 | 3.00 | 3.35E-02 | 0.67 | 2.56±0.81E+07 | 1.71±0.22E+07 |
| 4 | Citric acid | 1502.22 | 2.90 | 3.09E-11 | 3.23 | 2.50±0.37E+06 | 8.07±0.24E+06 |
| 5 | Alanine | 461.74 | 2.65 | 3.37E-02 | 0.38 | 1.10±0.66E+07 | 4.21±1.08E+06 |
| 6 | Tyrosine | 1578.13 | 2.32 | 3.89E-05 | 0.43 | 7.32±1.47E+06 | 3.13±0.13E+06 |
| 7 | Isoleucine | 757.67 | 1.98 | 2.42E-02 | 0.43 | 6.52±3.39E+06 | 2.83±0.36E+06 |
| 8 | Valine | 634.73 | 1.90 | 1.74E-02 | 0.48 | 6.47±2.85E+06 | 3.09±0.59E+06 |
| 9 | IMP | 2557.67 | 1.87 | 4.48E-03 | 4.02 | 9.90±5.61E+05 | 3.98±1.93E+06 |
| 10 | Phenylalanine | 1133.74 | 1.61 | 1.50E-03 | 0.55 | 4.30±1.03E+06 | 2.37±0.34E+06 |
| 11 | Galactonic acid | 1672.14 | 1.33 | 3.60E-03 | 1.83 | 1.81±0.25E+06 | 3.31±0.91E+06 |
| 12 | Malic acid | 1057.32 | 1.32 | 2.05E-04 | 1.20 | 6.74±0.53E+06 | 8.08±0.23E+06 |
| 13 | Myo-inositol | 1814.56 | 1.18 | 2.43E-07 | 0.54 | 2.14±0.19E+06 | 1.15±0.05E+06 |
